# Metabolic reprogramming in pulmonary fibrosis: mechanisms and therapeutic perspectives

**DOI:** 10.3389/fmed.2026.1982119

**Published:** 2026-10-22

**Authors:** Yaoqiao Wu, Xiaojing Su, Jingrun Zhou, Shaomao Zheng, Qiong Feng, Jingying Zhuang, Ziyi Yu, Hangqi Qiu, Yang Xia, Juanjuan Wu, Chengji Jin, Jing Wang

**Affiliations:** 1 Hainan Medical University, Haikou, Hainan, China; 2 Department of Respiratory Medicine, Second Affiliated Hospital of Hainan Medical University, Haikou, Hainan, China

**Keywords:** pulmonary fibrosis, metabolic reprogramming, pathogenesis, treatment strategies, signal transduction

## Abstract

Pulmonary fibrosis is a fatal disease characterized by progressive destruction of the lung parenchyma and excessive deposition of extracellular matrix, and is marked by a complex pathogenesis and limited treatment options. In recent years, metabolic reprogramming has emerged as a central mechanism by which cells adapt to stress and contribute to disease progression, attracting considerable interest within pulmonary fibrosis research. This review summarizes the metabolic reprogramming of key effector cells involved in pulmonary fibrosis, including lung fibroblasts, alveolar epithelial cells, macrophages, and endothelial cells, focusing on glucose, lipid, and amino acid metabolic pathways. It elucidates how these metabolic alterations drive fibrotic progression by influencing cell activation, proliferation, apoptosis, and intercellular communication. Building on these mechanistic insights, the review further summarizes potential therapeutic strategies targeting various metabolic pathways, aiming to establish a theoretical foundation and strategic directions for the development of novel precision therapies for pulmonary fibrosis. Furthermore, we highlight the metabolic communication among different pulmonary cell populations and discuss emerging challenges for precision metabolic therapies.

## 1 Introduction

Pulmonary fibrosis is a progressive lung disease characterized by the structural disruption of alveolar units and interstitial fibrosis, where the core pathological feature is the excessive deposition of extracellular matrix (ECM) caused by the abnormal activation and proliferation of fibroblasts (1). Despite recent advances in antifibrotic therapies, including pirfenidone and nintedanib, effective strategies capable of reversing fibrosis remain unavailable, highlighting the urgent need to identify novel therapeutic targets. Among them, the annual incidence of idiopathic pulmonary fibrosis (IPF) is rising steadily, currently 0.9–9.3/100,000 in Europe and North America, and 3.5–13.0/100,000 in Asia and South America, and the incidence of the disease increases significantly with age; the prevalence is higher in men than in women (2–4). The median survival of most patients is between 3 and 4 years, and the prognosis is poor (5). Although antifibrotic drugs such as pirfenidone and nintedanib have been adopted in clinical practice, they can only delay disease progression and cannot reverse pulmonary fibrosis; Furthermore, they are associated with side effects such as gastrointestinal disturbances and hepatotoxicity (6). Therefore, in-depth exploration of the pathogenesis of pulmonary fibrosis, the identification of new treatment targets, and the development of more effective therapeutic strategies have become key scientific issues that need to be addressed urgently. In recent years, metabolic reprogramming has emerged as an important adaptive response and a critical regulator of cellular behavior under fibrotic stress conditions. Studies have shown that key cell types in pulmonary fibrosis have undergone significant metabolic pattern alterations, involving abnormal changes in multiple pathways such as glucose metabolism, lipid metabolism, and amino acid metabolism (7), which profoundly drive the progression of fibrosis by affecting key pathological mechanisms such as autophagy, apoptosis, aging, and inflammatory responses. These metabolic alterations not only reflect cellular dysfunction but also actively reshape cellular phenotypes, including fibroblast activation, epithelial injury responses, and immune cell polarization within the fibrotic microenvironment. Therefore, in-depth analysis of the metabolic reprogramming characteristics of different cell types and the development of targeted therapy strategies accordingly have become a frontier area of research in pulmonary fibrosis. This article will summarize the current understanding of this topic, spanning from underlying mechanisms to therapeutic applications.

## 2 Overview and core mechanism of metabolic reprogramming in pulmonary fibrosis

### 2.1 Definition of metabolic reprogramming and its prevalence in fibrosis

Metabolic reprogramming refers to the process of fundamental changes in the energy metabolism pathway and biosynthetic mode of cells, enabling them to adapt to internal and external environmental pressures (8); This concept was first widely recognized in the field of oncology, known as the “Warburg effect”, which refers to cancer cells prioritizing glycolysis even under aerobic conditions to rapidly produce energy and biosynthetic precursors (9, 10). However, recent studies have revealed that metabolic reprogramming is also prevalent in fibrosis across multiple organs: for example, renal fibrosis is often accompanied by significant metabolic reprogramming, including alterations in fatty acid and glucose metabolism (11). The process of liver fibrosis involves metabolic adaptations of various cell types such as hepatic astrocytes, macrophages, and lymphocytes, and alterations in glucose and lipid metabolism resemble the “Warburg effect” observed in cancer cells (12). The core purpose of this reprogramming is to meet the rapidly increasing demands for energy and biosynthetic precursor required for cell proliferation, activation, extensive ECM secretion, and stress resistance (13, 14). In pulmonary fibrosis, metabolic reprogramming is not a single cellular event but encompasses interconnected changes affecting fibroblasts, alveolar epithelial cells, endothelial cells, and immune cells, which together constitute a pro-fibrotic metabolic microenvironment (7, 15, 16). This cross-organ universality suggests that metabolic reprogramming is a common and core pathophysiological feature of fibrotic diseases; therefore, understanding its universal mechanism of metabolic reprogramming has become a key entry point for elucidating the pathogenesis of pulmonary fibrosis and identifying new therapeutic targets.

### 2.2 Metabolic reprogramming drives the core pathological link of pulmonary fibrosis

The core pathological mechanisms of pulmonary fibrosis include fibroblast activation, epithelial-mesenchymal transition (EMT), mitochondrial dysfunction, oxidative stress, cellular senescence, and endoplasmic reticulum stress (17), it is still not entirely clear whether it is the cause of IPF or the metabolic adaptation resulting from fibrosis (18), and metabolic reprogramming plays a key regulatory role in these processes. First, metabolic reprogramming provides fibroblasts with sufficient energy and biosynthetic precursors by regulating key pathways, such as glycolysis and glutamine metabolism, thereby promoting their transformation into myofibroblasts and enhancing their proliferation, migration, and collagen synthesis (19, 20). Metabolic reprogramming also profoundly affects the fate of alveolar epithelial cells, which acquire stromal cell properties by undergoing the EMT process. This process not only compromises the structural integrity of the alveoli but also provides an additional source of fibroblasts for fibrotic lesions (21). Secondly, metabolic reprogramming is intricately linked to mitochondrial dysfunction and increased oxidative stress, which can further exacerbate alveolar epithelial cell damage and apoptosis, disrupt the integrity of alveolar structures, and create favorable conditions for the progression of fibrosis (22, 23). Ultimately, metabolic abnormalities influence ECM deposition and accelerate the progression of pulmonary fibrosis by impacting pathological processes such as cellular senescence, endoplasmic reticulum stress, and autophagy. Specifically, metabolic reprogramming leads to an imbalance in energy metabolism, triggering cellular senescence and the secretion of a large amount of profibrotic factors (24). The endoplasmic reticulum, as the main site for protein synthesis and folding, experiences a significantly enhanced stress response inducted by metabolic reprogramming, resulting in the accumulation of unfolded or misfolded proteins that cause cell apoptosis and the progression of pulmonary fibrosis (24). Metabolic abnormalities can also lead to impaired autophagy function, resulting in the accumulation of intracellular debris and further exacerbating cell damage and pulmonary fibrosis (25). In summary, metabolic reprogramming profoundly drives the core pathological mechanisms of pulmonary fibrosis through multi-pathway and multi-level regulation, making it a key factor in the pathogenesis of pulmonary fibrosis.

## 3 Metabolic reprogramming of lung fibroblasts

### 3.1 Reprogramming of glucose metabolism in lung fibroblasts

In the process of pulmonary fibrosis, abnormal glucose metabolism in lung fibroblasts has been identified as a key initiator driving fibroblast activation and extracellular matrix deposition, in which the core of abnormal glucose metabolism is aerobic glycolysis (i.e., the Warburg effect). It was found that activated lung fibroblasts upregulated PFKFB3 to drive aerobic glycolysis and stabilize hypoxia-inducible factor 1-alpha (HIF-1*α*), which directly induced *α*-SMA expression. Knockout of PFKFB3 by CRISPR-Cas9 or by using its small molecule inhibitor 3PO can effectively block this differentiation process and ameliorate pulmonary fibrosis in mice (26). These findings establish glycolytic reprogramming as a core metabolic feature in the pathological process of pulmonary fibrosis for the first time, providing a theoretical basis for targeting PFKFB3 to intervene in the metabolic state of lung fibroblasts. Another study further revealed the transcriptional regulation mechanism of PFKFB3: the RNA-binding protein PCBP3 promotes glycolytic reprogramming by promoting PFKFB3 transcription, while anlotinib inhibits PFKFB3 transcriptional translation by downregulating PCBP3, thereby blocking myofibroblast activation and pulmonary fibrosis (27). These results collectively indicate that aerobic glycolysis plays a key role in the pathogenesis of pulmonary fibrosis. Furthermore, it has been confirmed that the activation of glycolysis dependent on glucose transporter type 1 (GLUT1) is an important driving factor for age-related pulmonary fibrosis; GLUT1 promotes lung fibroblast activation by regulating the MAPK pathway, and knockdown of GLUT1 expression effectively blocks myofibroblast differentiation and bleomycin-induced pulmonary fibrosis in mice (28). Dihydrogenomaritin has also been confirmed to down-regulate the expression of GLUT1 by regulating the STAT3/p-STAT3 signaling pathway, thereby inhibiting the activation of cell proliferation and migration caused by glycolysis enhancement in fibroblasts, and demonstrating anti-fibrotic effects in a bleomycin-induced mouse pulmonary fibrosis model (29). The above two studies jointly confirmed that GLUT1-dependent glycolysis is a key metabolic node in the activation of fibroblasts in the process of pulmonary fibrosis, and targeting GLUT1 or its upstream regulators (such as STAT3) has significant anti-fibrotic potential. In addition, lipopolysaccharide (LPS) has been shown to induce aerobic glycolysis in lung fibroblasts and promote collagen synthesis by activating the PI3K-Akt-mTOR signaling pathway to upregulate PFKFB3 expression, an effect that can be significantly reversed by both the PI3K-Akt inhibitor LY294002 and the mTOR inhibitor rapamycin (30). Tanshinone IIA can downregulate the expression of hexokinase 2 (HK2) and inhibit ACLY activity by inhibiting the PI3K/Akt/mTOR/HIF-α pathway and inhibiting ACLY activity, thereby blocking glycolysis-mediated collagen synthesis and reversing myofibroblast activation (31). These two studies confirm each other from the perspectives of forward activation and reverse inhibition, and jointly reveal the central regulatory position of the PI3K-Akt-mTOR signaling axis in the glycolytic reprogramming of lung fibroblasts. Finally, different regulators showed a two-way balance of “positive promotion and negative inhibition” on the glycolytic regulation of lung fibroblasts. SIK2, as a key molecule in positive regulation, is markedly overexpressed in activated fibroblasts in patients with pulmonary fibrosis and model mice, and enhances glycolysis by upregulating the expression of glycolysis-related enzymes (HK2, PFKFB3, PKM2, GLUT1, LDHA and PGAM1), thereby promoting fibroblast activation, proliferation and migration (32). Similarly, circHIPK3 is highly expressed under the induction of TGF-*β*1, which relieves the inhibition of the glycolytic transcription factor FOXK2 by sequestration of miR-30a-3p, thereby indirectly enhancing glycolysis and promoting cell activation and fibrosis progression (33). The integrin *β*3-PKM2 pathway specifically mediates enhanced glycolysis in lung fibroblasts induced by mechanical ventilation, and targeting this pathway can improve mechanical ventilation-associated pulmonary fibrosis (34). CPSF6 and vitamin D played a negative regulatory role. CPSF6 is down-regulated in fibrotic lung tissue, and its deletion generates a short 3'UTR LDHA isoform through alternative polyadenylylation, circumventing the targeted inhibition of miR-4317, resulting in LDHA expression upregulation and enhanced glycolysis, which aggravates silica-induced pulmonary fibrosis in mice (35); Vitamin D inhibits the expression of HK2, a key enzyme for glycolysis, and blocks fibroblast activation and abnormal glucose metabolism by negatively regulating p-STAT3 levels (36). In summary, pulmonary fibroblast activation is closely related to the progression of pulmonary fibrosis, and its core metabolic feature is reprogramming to aerobic glycolysis. Several key molecules, such as PFKFB3, GLUT1, HK2, and PKM2, have been identified as important metabolic nodes driving this process, and the PI3K-Akt-mTOR signaling axis is in the core upstream regulatory position. In addition, the study revealed a complex regulatory network composed of positive factors such as SIK2 and circHIPK3 and negative factors such as CPSF6 and vitamin D, which jointly controlled the level of glycolysis. Therefore, we believe that future treatment strategies should break through the limitations of a single target and focus on reshaping the systemic balance of metabolic regulatory networks, which will provide more potential intervention directions for overcoming pulmonary fibrosis (Figure 1).

**Figure 1:**
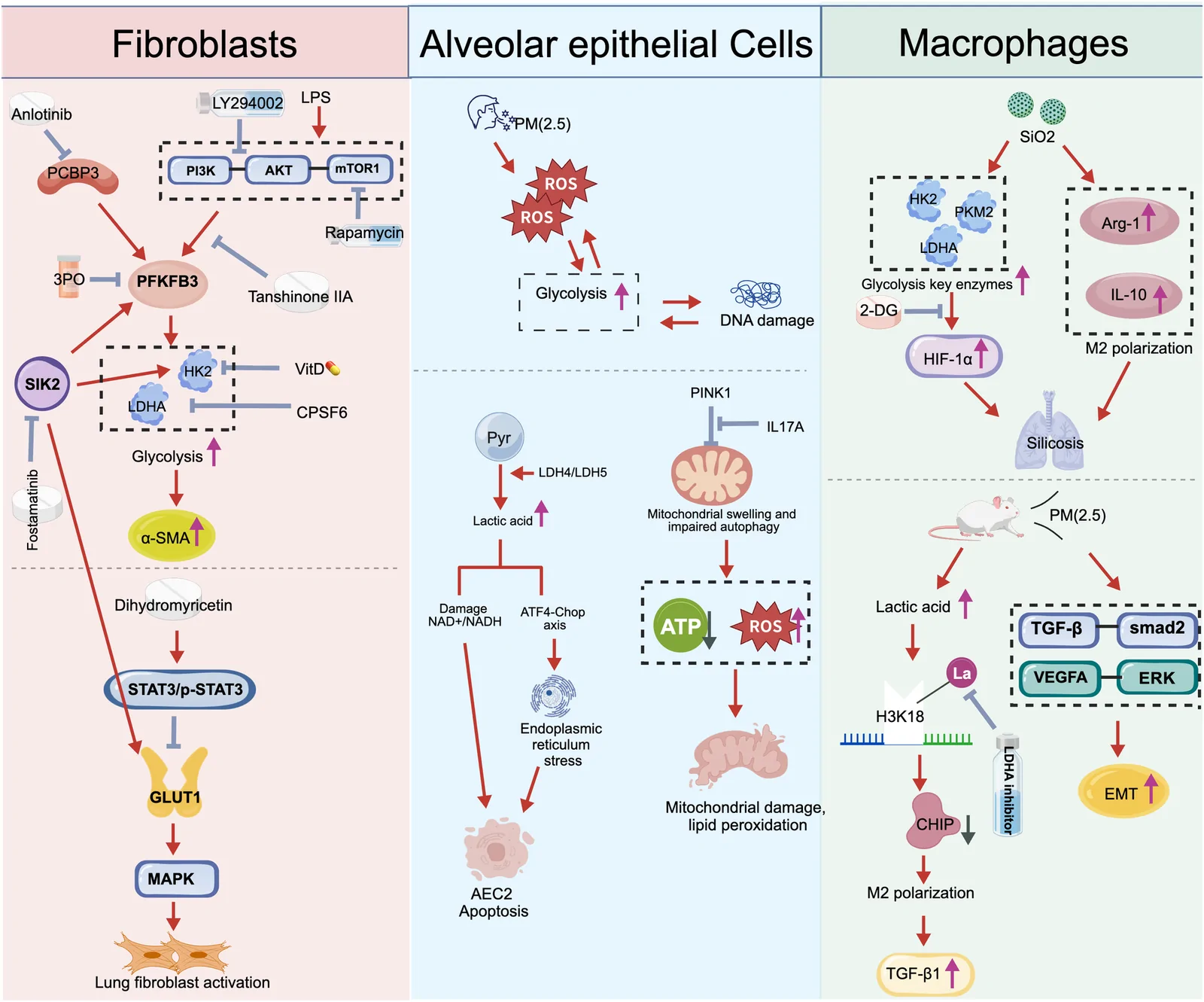
Glycolytic metabolic reprogramming in pulmonary fibrosis. Glycolytic metabolic reprogramming contributes to pulmonary fibrosis by regulating fibroblast activation, alveolar epithelial cell injury, and macrophage polarization. **(A)** Fibroblasts. Multiple regulatory pathways, including PI3K/AKT/mTORC1, SIK2, PFKFB3, HK2, LDHA, STAT3, and GLUT1 signaling, enhance glycolytic activity and promote *α*-SMA expression and lung fibroblast activation. **(B)** Alveolar epithelial cells. PM2.5-associated metabolic stress and enhanced glycolysis are linked to ROS accumulation and DNA damage. Increased lactate production, impaired NAD+/NADH homeostasis, ATF4-CHOP-mediated endoplasmic reticulum stress, and PINK1-related mitochondrial dysfunction further contribute to AEC2 apoptosis, mitochondrial damage, and lipid peroxidation. **(C)** Macrophages. SiO2-induced glycolytic activation involving HK2, PKM2, LDHA, and HIF-1*α*, together with increased Arg-1 and IL-10 expression, promotes M2 macrophage polarization and contributes to silicosis. PM2.5-associated lactate accumulation and histone lactylation at H3K18 are also associated with M2 polarization and activation of TGF-*β*/SMAD2 and VEGFA/ERK signaling. Created with BioGDP.com (107).

### 3.2 Reprogramming of lipid metabolism in lung fibroblasts

Compared with glucose metabolism, the role of lipid metabolism in pulmonary fibrosis has been relatively little studied. Previous studies have shown that lipid metabolism reprogramming is also a key regulatory link in the activation and functional transition of lung fibroblasts (7). Lipids not only serve as cell membrane structure and energy sources, but also mediate signal transduction through their active metabolites, affecting fibroblast proliferation, activation, and ECM synthesis. First, the abnormal regulation of prostaglandin signaling molecules is an important feature of lipid metabolism reprogramming in lung fibroblasts, and prostaglandin F2*α* (PGF2 *α*) can directly stimulate lung fibroblast proliferation and collagen production through its receptors, and receptor deletion, which is associated with the inhibition of TGF-*β*1 signaling, exhibits a synergistic effect that ameliorates pulmonary fibrosis (37), suggesting that the PGF2*α* pathway is independent of classical TGF-*β*1pro-fibrotic pathways of the pathway. Prostaglandin E synthase (PTGES) is abnormally upregulated under inflammation-induced conditions and promotes myofibroblast differentiation by activating the AKT-HIF-1 *α*-dependent glycolytic pathway, and its inhibitors can reverse this process, revealing a novel mechanism of cross-regulation of lipid and glucose metabolism (38). Secondly, lysophosphatidic acid (LPA) is a small-molecule lipid ubiquitous in living organisms (39), and the pathway mediated by LPA and its receptors is key to connecting lung injury and fibrosis. Studies in bleomycin-induced models have shown elevated LPA levels in alveolar lavage fluid. Both LPA1 and LPA2 receptors are implicated in fibrosis: LPA1 drives fibrosis by mediating fibroblast recruitment and vascular leakage (40), whereas LPA2 promotes myofibroblast differentiation by regulating TGF-*β*1 expression, with LPA2 gene knockout alleviating pulmonary fibrosis (41). Furthermore, imbalances in fatty acid metabolism have also been shown to be involved in pulmonary fibrosis. In silica-induced pulmonary fibrosis, myofibroblasts exhibit significant metabolic switching features with inhibition of fatty acid oxidation and enhanced glycolysis, and this HIF-1 *α*-driven conversion leads to increased ECM deposition and decreased degradation (42). Impaired fatty acid oxidation can lead to the accumulation of lipid intermediate products (such as diacylglycerol and ceramides) within the cells and activate the pro-fibrotic TGF-*β*1 signaling pathway as important signaling molecules, thereby forming a positive feedback loop that continuously drives the process of lung fibrosis (43); while in aging-related pulmonary fibrosis, impaired fatty acid oxidation is caused by the highly expressed UCP2 (44); Drp1-mediated mitochondrial division regulates the reprogramming of fibroblast lipid metabolism through the ROS/HIF-1*α* pathway, promoting the progression of pulmonary fibrosis (45), it can be seen that abnormal fatty acid metabolism is closely related to changes in mitochondrial dynamics. Finally, lipid metabolism reprogramming also involves abnormalities in lipid storage and utilization, with lipophagy being a form of selective autophagy responsible for degrading lipid droplets to provide energy and metabolic precursors; Cigarette smoke exposure increases mitochondrial ROS by reducing SIRT1 expression, disrupting autophagic flux to reduce lipophagy, leading to increased lipid content in fibroblasts and promoting fibroblast proliferation and activation (46). This finding sheds light on how environmental factors contribute to the development of pulmonary fibrosis by influencing lipid metabolism reprogramming. In conclusion, lipid metabolism reprogramming synergistically drives the activation and fibrosis of lung fibroblasts through multiple mechanisms such as prostaglandin signal imbalance, lysophosphatidic acid receptor activation, fatty acid oxidation inhibition and lipophagy disorders. These findings reveal the key role of lipid metabolic homeostasis disruption in the pathogenesis of pulmonary fibrosis, suggesting that multi-target intervention strategies aimed at lipid metabolism pathways may provide a new breakthrough in anti-fibrosis therapy (Figure 2).

**Figure 2:**
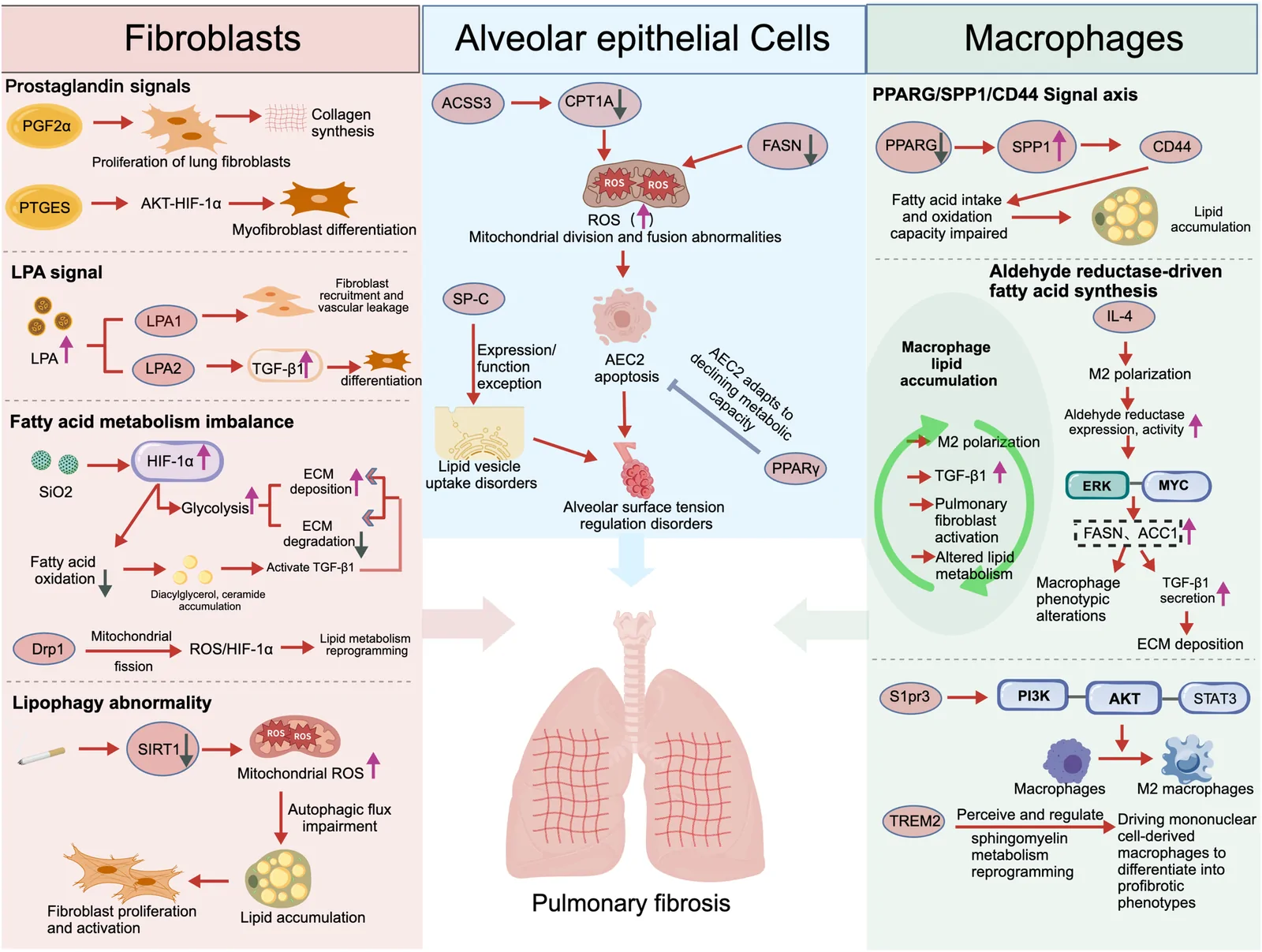
Lipid metabolic reprogramming in pulmonary fibrosis. Lipid metabolic dysregulation contributes to pulmonary fibrosis by altering fibroblast activation, alveolar epithelial cell homeostasis, and macrophage function. **(A)** Fibroblasts. Dysregulated prostaglandin and lysophosphatidic acid (LPA) signaling promotes fibroblast proliferation, myofibroblast differentiation, and TGF-*β*1-related responses. Altered fatty acid metabolism, glycolysis, mitochondrial ROS production, and lipophagy are associated with lipid accumulation, extracellular matrix dysregulation, and fibroblast activation. **(B)** Alveolar epithelial cells. Alterations in ACSS3, CPT1A, and FASN are associated with mitochondrial division and fusion abnormalities, ROS accumulation, lipid vesicle uptake disorders, alveolar surface tension dysregulation, and AEC2 apoptosis. **(C)** Macrophages. Dysregulated lipid uptake and metabolism involving the PPARG/SPP1/CD44 signaling axis contribute to impaired fatty acid utilization and lipid accumulation. Aldehyde reductase-driven fatty acid synthesis promotes M2 polarization, increases FASN/ACC1 expression and TGF-*β*1 secretion, and contributes to extracellular matrix deposition. S1PR3-PI3K/AKT/STAT3 and TREM2-associated sphingomyelin metabolic reprogramming further promote profibrotic macrophage phenotypes. Created with BioGDP.com (107).

### 3.3 Reprogramming of amino acid metabolism in lung fibroblasts

Abnormal activation of lung fibroblasts and excessive deposition of ECM are core features of the pathological process of pulmonary fibrosis, and collagen, as the main component of ECM, is highly dependent on the supply of specific amino acids, including glycine, proline, and glutamine (47). As the most abundant amino acid in collagen, glycine's endogenous supply is crucial. TGF-*β*1 activates the PI3K-Akt-mTORC1 signaling pathway, promoting the accumulation of the transcription factor ATF4, and thereby upregulating the expression of key enzymes in the serine-glycine synthesis pathway. This finding explains why the use of partial mTORC1 inhibitors such as rapamycin alone is ineffective, while completely blocking the kinase activity of mTORC1 can effectively inhibit the metabolic reprogramming induced by ATF4 (48). Further studies have found that ATF4 maintains amino acid homeostasis in lung fibroblasts by regulating enzymes involved in amino acid homeostasis and transporting aminoacyl-tRNA synthase, and inhibiting mTOR not only leads to loss of ATF4 target gene expression, but also reduces the expression of glycolytic enzymes and mitochondrial electron transport chain subunits (49). Proline metabolic reprogramming also plays a crucial role in pulmonary fibrosis, and its associated metabolic disorders are closely linked to oxidative stress signaling pathways. NOX4 can mediate the activation of the ARG1/OAT pathway and regulate the conversion of arginine to proline, thereby promoting the activation of TGF-*β*/Smad signaling pathway and collagen deposition (50). Proline synthesis is also regulated by the mechanical microenvironment. Kindlin-2 is translocated to mitochondria and forms a complex with PYCR1, a key enzyme for proline synthesis, which significantly increases the level of proline synthesis, promotes lung fibroblast activation and collagen matrix deposition. Furthermore, knockout of Kindlin-2 or the use of pirfenidone and nintedanib can effectively inhibit the formation of this complex and reverse abnormal proline metabolism (51, 52). It can be seen that proline synthesis is a key metabolic node connecting the mechanical environment, oxidative stress and collagen synthesis. The reconfiguration of glutamine metabolism is another important feature of the metabolic reprogramming of lung fibroblasts. Previous studies have suggested that glutamine is decomposed into *α*-ketoglutarate, allowing it to enter the tricarboxylic acid cycle and providing cells with energy and biosynthetic intermediates (53); However, the primary function of glutamine metabolism in pulmonary fibrosis is to support amino acid biosynthesis rather than energy production, which is significantly different from the glutamine metabolism pattern in tumor cells (54, 55). TGF-*β*1 can enhance the conversion of glutamine to glutamate by activating SMAD3 and MAPK pathways to regulate the expression of glutaminase isoform GLS1, providing energy and metabolic precursors for myofibroblast differentiation, and extracellular glutamine depletion or GLS1 silencing can directly block TGF-*β*1-induced fibroblast activation (56). Tanshinone IIA can reduce the metabolic flux of glutamine in mitochondria by inhibiting GLS1 expression, and simultaneously target and inhibit the expression of key enzymes in proline synthesis, thereby achieving bidirectional regulation of glutamine metabolic reprogramming and inhibiting collagen synthesis (20). In conclusion, the amino acid metabolism reprogramming of lung fibroblasts is synergistically regulated by multiple metabolic pathways involving glycine, proline and glutamine to jointly support collagen synthesis and fibroblast activation. These metabolic pathways are closely coupled with TGF-*β*1 signaling, the mechanical microenvironment and oxidative stress to form a complex regulatory network (Figure 3).

**Figure 3:**
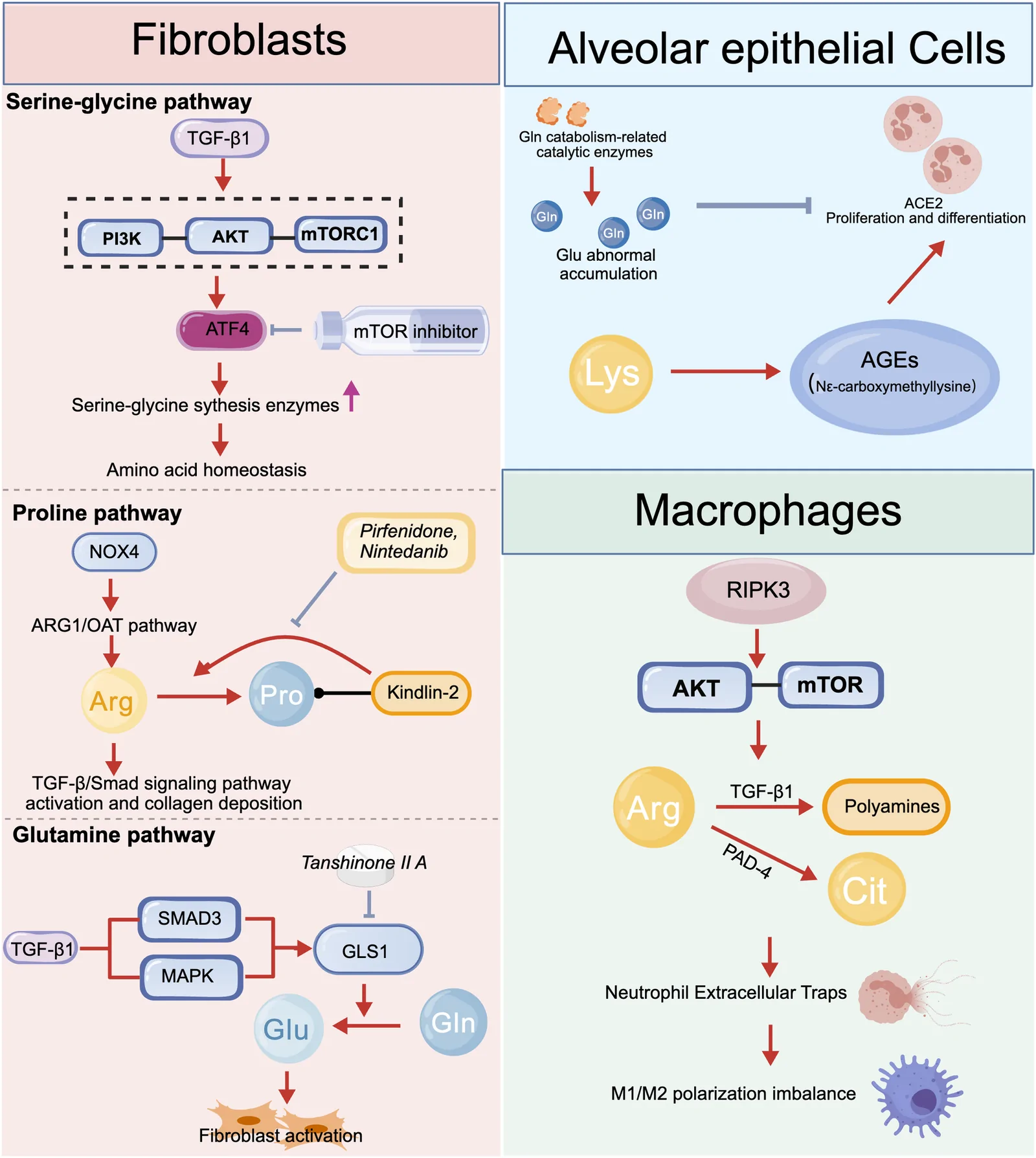
Amino acid metabolic reprogramming in pulmonary fibrosis. Amino acid metabolic remodeling regulates fibroblast activation, alveolar epithelial cell function, and macrophage polarization during pulmonary fibrosis. **(A)** Fibroblasts. TGF-*β*1-associated PI3K/AKT/mTORC1-ATF4 signaling promotes serine-glycine synthesis and amino acid metabolic remodeling. Alterations in the NOX4-ARG1/OAT pathway and proline metabolism contribute to TGF-*β*/SMAD-associated collagen deposition, while TGF-*β*1/SMAD3/MAPK signaling regulates GLS1-dependent glutamine metabolism and fibroblast activation. **(B)** Alveolar epithelial cells. Altered glutamine catabolism and abnormal glutamate accumulation are associated with impaired AEC2 proliferation and differentiation. lysine-derived AGEs (N*ε*-carboxymethyl lysine) is also associated with regulation of AEC2 proliferation and differentiation. **(C)** Macrophages. RIPK3-associated AKT/mTOR signaling regulates arginine metabolism, with downstream effects on TGF-*β*1-associated polyamine synthesis and PAD4-mediated citrullination. These metabolic and inflammatory alterations contribute to neutrophil extracellular trap formation and M1/M2 macrophage polarization imbalance. Created with BioGDP.com (107).

## 4 Metabolic reprogramming of alveolar epithelial cells

### 4.1 Reprogramming of glucose metabolism in alveolar epithelial cells

Alveolar Epithelial Cells (AECs) play a central role in pulmonary fibrosis, exhibiting significant glucose metabolism reprogramming characterized by aberrant glycolytic activation and mitochondrial dysfunction, particularly notable in idiopathic pulmonary fibrosis (IPF) (57). Persistent glycolytic activation is closely linked to AEC senescence and epithelial-mesenchymal transition (EMT), forming a bidirectional regulatory loop: senescent AECs display heightened glycolysis and impaired oxidative phosphorylation, while sustained glycolysis exacerbates cellular senescence through excessive reactive oxygen species (ROS) production and DNA damage (58). This self-perpetuating cycle impairs epithelial regeneration and accelerates fibrosis. In PM_2.5_ exposure-induced pulmonary fibrosis, reactive oxygen species (ROS)-driven pseudohypoxia signaling activates HIF-1*α*, thereby promoting glycolytic metabolic transitions and EMT in alveolar epithelial cells (59). Secondly, type II alveolar epithelial cells (AEC2) are the core effector cells of glucose metabolism reprogramming, and their metabolic phenotypic transformation is mediated by LDH-specific upregulation: pulmonary fibrosis-associated AEC2 mainly express LDH4/LDH5 isoforms that promote pyruvate to lactate conversion, while non-fibrotic AEC2 cells predominantly express LDH2/LDH3 isoforms (60). In the process of pulmonary fibrosis, this balance is disrupted, and the lactate level in the fibrotic lungs increases 3-fold compared to healthy tissue. The mechanism is closely related to the overproduction and clearance of lactate (61). Excessive accumulation of lactate is not an inert metabolic waste product, but a key mediator driving fibrosis, which accelerates AEC2 cell senescence by disrupting the NAD+/NADH redox balance (62), and by activating endoplasmic reticulum stress through the ATF4-Chop axis, ultimately triggering AEC2 apoptosis (63). Mitochondrial dysfunction is another core feature and upstream driver of glucose metabolic reprogramming in AEC2. In pulmonary fibrosis, AEC2 manifests as swelling of mitochondrial structures and impaired mitochondrial autophagy, resulting in decreased oxidative phosphorylation efficiency and insufficient ATP production, while a large amount of mitochondrial ROS is produced, which exacerbates mitochondrial DNA damage and lipid peroxidation, forming a vicious cycle of “metabolic disorder-oxidative stress” (57, 64). The PINK1/Parkin-mediated mitochondrial autophagy pathway plays a key role in this process (65), while IL-17A induces mitochondrial dysfunction in alveolar type II epithelial cells by inhibiting PINK1-mediated mitochondrial autophagy, thereby exerting its pro-fibrotic effect (66). Finally, abnormal activation of glycolysis and mitochondrial dysfunction do not occur independently. Studies have found that damaged alveolar epithelial cells are often accompanied by mitochondrial dysfunction, resulting in decreased oxidative phosphorylation efficiency and insufficient energy production, which in turn prompts cells to switch to glycolysis to maintain basic metabolic needs (67). In summary, alveolar epithelial cell glucose metabolism reprogramming drives cell senescence, apoptosis and EMT through the vicious cycle of abnormal glycolytic activation and mitochondrial dysfunction, and lactate plays a central role as a key metabolic mediator in it, suggesting that targeting the key nodes of glucose metabolism may break this cycle and provide a new direction for pulmonary fibrosis treatment.

### 4.2 Reprogramming of lipid metabolism in alveolar epithelial cells

Alveolar epithelial cells are the main synthesis sites for lung surfactants, and their homeostasis of lipid metabolism is essential for maintaining alveolar structure and function (68). Lipid metabolism in alveolar epithelial cells is significantly disturbed, which directly affects surfactant synthesis and homeostasis, leading to alveolar instability and repeated microdamage in pulmonary fibrosis (69). Mitochondrial dysfunction and oxidative stress are the core links of lipid metabolism disorders in alveolar epithelial cells. It was found that ACSS3 reduces fatty acid oxidation and enhances anaerobic glycolysis by inhibiting CPT1A expression, and this metabolic reprogramming leads to alveolar epithelial cell function inactivation by reducing mitochondrial division and fusion, increasing ROS production, inhibiting mitochondrial autophagy, and promoting apoptosis (70). Fatty acid synthase expression is significantly reduced in the lung tissue of patients with idiopathic pulmonary fibrosis and mice with bleomycin-induced fibrosis, and fatty acid synthase overexpression can markedly inhibit bleomycin-induced alveolar epithelial cell death by reducing mitochondrial membrane potential loss and mitochondrial reactive oxygen species production (71). Secondly, abnormal regulation of lipid metabolism-related signaling pathways further exacerbates the process of fibrosis. PPAR-*γ*, as a lipid stress-related factor, is down-regulated in pulmonary fibrosis and affects the metabolic adaptability of alveolar epithelial cells (67); In mTOR inhibitor-induced mouse lung injury models, alveolar epithelial cells exhibited lipid metabolism stress, manifested by increased cholesterol and free fatty acid levels, accompanied by downregulation of PPAR-*γ* expression (72). In addition, in pulmonary fibrosis, surfactant proteins such as SP-C have abnormal expression and function, and further affect alveolar surface lipid composition and surface tension regulation by modulating lipid vesicle uptake (73). Finally, single-cell sequencing data also showed that the differentially expressed genes of lipid metabolism in AEC2 were mainly related to the cytidine diphosphate-diacylglycerol pathway, cholesterol metabolism, and triglyceride synthesis (74). In summary, lipid metabolism reprogramming in alveolar epithelial cells plays a key role in the pathogenesis of pulmonary fibrosis, involving mitochondrial function, surfactant metabolism, and signaling pathway regulation. An in-depth understanding of these metabolic changes will not only help elucidate the pathogenesis of pulmonary fibrosis but also provide a theoretical basis for the development of new therapeutic strategies.

### 4.3 Reprogramming of amino acid metabolism in alveolar epithelial cells

During pulmonary fibrosis, amino acid metabolism reprogramming in alveolar epithelial cells exhibits multi-dimensional and multi-level characteristics. Glutamine metabolism, as an important pathway for energy metabolism and biosynthesis of alveolar epithelial cells, shows significant abnormalities in pulmonary fibrosis. Studies have shown that the expression level of glutamine catabolism-related catalytic enzymes in alveolar type II epithelial cells of IPF patients is significantly down-regulated, leading to abnormal accumulation of unutilized glutamine in cells, and this metabolic disorder directly affects the proliferation and differentiation of alveolar epithelial cells (75); There is also evidence to support that glutamine supplementation increases intracellular intermediate metabolite levels and reduces DNA damage and cell death, suggesting that epithelial cells may maintain their own stability under injury stress by switching to glutamine utilization (76). The arginine metabolic pathway is also altered in alveolar epithelial cells during pulmonary fibrosis. As arginine serves as a substrate for nitric oxide synthase, disturbances in its metabolism may affect the oxidative stress status and inflammatory response of these cells. At the same time, lysine-derived advanced glycation end products (AGEs) such as N*ε*-carboxymethyl lysine play an important role in cell proliferation and differentiation, and their metabolic abnormalities may further exacerbate alveolar epithelial cell damage and abnormal repair (77). Metabolomics studies further reveal circadian changes in amino acid metabolism in pulmonary fibrosis. It was found that amino acid metabolism showed significant circadian oscillations in normal mouse lung tissue, while in IPF mice, although the amino acid metabolism rhythm pattern remained basically unchanged, its metabolic level was significantly up-regulated, especially in the level of urea cycle-related metabolites, which were significantly increased in IPF lung tissue (78). It can be seen that by regulating specific amino acid metabolism pathways, such as enhancing glutamine metabolism to promote alveolar epithelial cell regeneration or inhibiting abnormal arginine metabolism to reduce oxidative damage, these therapeutic strategies may become new directions for the treatment of pulmonary fibrosis.

## 5 Metabolic reprogramming of macrophages

### 5.1 Macrophage glucose metabolism reprogramming

In pulmonary fibrosis, the reprogramming of glucose metabolism by macrophages presents unique characteristics. Studies have shown that in the silica-induced pulmonary fibrosis model, the expression of key glycolytic enzymes such as HK2, PKM2, and LDHA is significantly increased, and the expression of macrophage M2 activation markers such as Arg-1 and IL-10 is also correspondingly up-regulated (79). Similarly, alveolar macrophages exhibit a significant pro-fibrotic M2-like phenotype in fibrotic mouse lung tissue, which is closely related to enhanced glycolysis, unlike classical IL-4-induced M2 macrophages that rely on fatty acid oxidation or lipolysis (80). Another study found that HIF-1*α* plays a key regulatory role in macrophage glucose metabolism reprogramming. In mouse models of silicosis, HIF-1*α* and glycolysis-related gene expression were up-regulated, and 2-DG, a glycolytic inhibitor, reduced the transcription of the inflammatory cytokine IL-1β by inhibiting HIF-1*α* expression, thereby attenuating lung inflammation and fibrosis (81). Lactic acid, a glycolysis product, plays multiple roles in pulmonary fibrosis, and lactate not only acts as an intermediate product of energy metabolism, but also affects gene expression through histone lactylation. In a PM_2.5_-induced mouse model of pulmonary fibrosis, increased lactate production by macrophages inhibited the expression of carboxyl terminus of Hsc70-interacting protein (CHIP, encoded by Stub1) expression by promoting lactylation at the H3K18 site within the Stub1 promoter region; this facilitated M2 macrophage polarization and increased TGF-*β*1 secretion, thereby exacerbating pulmonary fibrosis (82). Furthermore, this is a strong link between macrophage glucose metabolism reprogramming and epigenetic modifications. As an m6A reader, IGF2BP1 can promote TLR4-mediated macrophage M2 polarization and glycolytic activation by stabilizing THBS1 mRNA, driving pulmonary fibrosis progression, revealing the key role of the IGF2BP1/THBS1/TLR4 regulator*y* axis in pulmonary fibrosis (83). In addition to regulating cellular polarization states, macrophage metabolic reprogramming can also affect their interactions with other cells. Macrophage glycolysis and secretion of profibrotic cytokines by PM_2.5_ treatment induce EMT in epithelial cells by activating TGF-*β*/Smad2 and VEGFA/ERK pathways. In contrast, LDHA inhibitor pretreatment can effectively alleviate PM_2.5_-induced lung inflammation and fibrosis in mice by inhibiting glycolysis and histone lactation modification (84). Metabolic interactions between macrophages and fibroblasts are also significant in pulmonary fibrosis. LPS-induced activation of the lung macrophage JNK signaling pathway and TNF-α secretion promote aerobic glycolysis and lactate production in lung fibroblasts, and this inflammatory-metabolic interaction plays a key role in LPS-induced pulmonary fibrosis (85). Other studies have found that the TREK-1 potassium channel drives pulmonary fibrosis through cell interaction mechanisms: on the one hand, upregulation of TREK-1 expression in macrophages promotes their polarization to the pro-fibrotic M2 phenotype, which in turn enhances the paracrine activation of fibroblasts; On the other hand, TREK-1 in fibroblasts directly regulates their differentiation into myofibroblasts through the FAK/p38/YAP signaling pathway (86). In conclusion, macrophage glucose metabolism reprogramming in pulmonary fibrosis is a complex regulatory network involving multiple signaling pathways, epigenetic mechanisms, and cell interactions. An in-depth understanding of this process will not only help elucidate the pathogenesis of pulmonary fibrosis, but also suggest that intervention targeting key nodes of macrophage glucose metabolism may be an effective strategy for treating pulmonary fibrosis.

### 5.2 Macrophage lipid and amino acid metabolism reprogramming

In the pathological process of pulmonary fibrosis, macrophages, as key immunoregulatory cells, not only modulate their own functional state, but also promote the process of fibrosis through various mechanisms (7). Single-cell sequencing analysis showed that macrophages were the most active cell type in IPF, and almost all lipid metabolism-related genes were significantly altered in IPF macrophages (74). The expression of PPARG, a key transcription factor for lipid metabolism, was significantly down-regulated in alveolar macrophages from patients with IPF, whereas SPP1 the expression was up-regulated; this forms the PPARG/SPP1/CD44 signaling pathway, leading to impaired fatty acid uptake and oxidative capacity, and ultimately promoting abnormal lipid accumulation in macrophages and pulmonary fibrosis (87). In models of IL-4-induced M2 macrophage polarization, aldose reductase expression and activity are significantly elevated; concurrently, activation of the ERK-MYC signaling pathway upregulates FASN and ACC1, thereby enhancing fatty acid synthesis. This metabolic reprogramming not only alters the macrophage phenotype but also activates lung fibroblasts by secreting TGF-*β*1, promoting extracellular matrix deposition (88). Furthermore, S1pr3, a lipid signaling receptor, plays a pivotal role in macrophage lipid metabolic reprogramming; specifically, during pulmonary fibrosis, S1pr3 drives polarization toward the M2 pro-fibrotic phenotype via activation of the PI3K/Akt-Stat3 signaling pathway. Targeted inhibition of S1pr3 can mitigate bleomycin-induced pulmonary fibrosis by intervening in lipid-mediated signaling (89). As a surface lipid receptor of macrophages, TREM2 drives the differentiation of monocyte-derived macrophages into pro-fibrotic phenotypes by sensing and regulating sphingolipid metabolism reprogramming; targeting TREM2 can alleviate pulmonary fibrosis in mice by intervening in macrophage sphingomyelin synthesis and chemokine CCL2 secretion, thereby remodeling the lipid metabolism microenvironment in the lungs (90). Macrophages also undergo significant amino acid metabolic reprogramming in the pulmonary fibrosis microenvironment. RIPK3 drives amino acid metabolic reprogramming in scar-associated macrophages via the AKT-mTOR pathway, promoting the TGF-*β*1-mediated conversion of arginine to polyamines. Consequently, targeting RIPK3 inhibits polyamine accumulation and associated pro-fibrotic effects by blocking this metabolic axis (91). PAD-4 mediates the citrullination of arginine residues, drives the release of neutrophil extracellular traps (NETs), and subsequently induces an imbalance in macrophage M1/M2 polarization. Inhibition of PAD-4 activity can simultaneously reduce the synergistic release of M1 pro-inflammatory and M2 pro-fibrotic cytokines, thereby exerting a dual anti-fibrotic effect in the pulmonary fibrosis model (92). During the process of pulmonary fibrosis, macrophages jointly shape their pro-fibrotic phenotype and regulate fibroblast activation through lipid and amino acid metabolic reprogramming, and these abnormalities of metabolic nodes constitute an important mechanism for the pathogenesis of pulmonary fibrosis. Experiments show that some metabolic changes show inconsistency between mouse models and IPF patients, so there is still insufficient evidence to consider metabolic reprogramming as the main cause of IPF (93).

## 6 Metabolic reprogramming of endothelial cells

The metabolic reprogramming of endothelial cells also plays an important role in the process of pulmonary fibrosis, mainly involving the shift of glucose metabolism and lipid homeostasis imbalance. In terms of glucose metabolism, pulmonary microvascular endothelial cells rely on LDHA-mediated aerobic glycolysis to maintain their rapid proliferation (94). It is worth noting that the ROCK2 inhibitor KD025 can significantly inhibit glycolysis in lung microvascular endothelial cells, promote oxidative phosphorylation, reduce intracellular pH, inhibit cell migration, and ultimately enhance lung barrier integrity (95). This suggests that shifting endothelial cell metabolic patterns from glycolysis to oxidative phosphorylation through pharmacological interventions may be a potential strategy to protect the alveolar-capillary barrier. Lipid metabolism disorders are another key factor driving endothelial dysfunction. Single-cell sequencing analysis revealed that endothelial cells had abnormal reprogramming of sphingomyelin and phospholipid metabolism genes in fibrosis (74). Mechanistically, endothelium-specific S1PR1 deletion disrupts lipid signaling, leading to increased vascular permeability and exacerbation of fibrosis (96). However, abnormal LDL accumulation caused by LDLR dysfunction directly induces endothelial apoptosis and pro-fibrotic phenotypic transformation (97). Interventions targeting lipid metabolism, such as the application of VEGF inhibitors or restoration of LDLR function, have shown potential in alleviating fibrosis (97, 98). In conclusion, endothelial cell metabolic reprogramming, encompassing disturbances in the glycolytic-oxidative phosphorylation balance and lipid metabolism, contributes to pulmonary fibrosis by affecting endothelial cell survival and barrier function. Targeting endothelial cell-specific metabolic nodes, such as regulating energy metabolism or restoring LDLR function, is expected to offer novel therapeutic avenues for treating pulmonary fibrosis (Table 1).

**Table 1:** Metabolic-targeted therapeutic strategies for pulmonary fibrosis.

Cell type	Metabolic pathway	Key molecules/pathways	Functional consequences
Fibroblasts	Glucose metabolism	PCBP3, PFKFB3, GLUT1, STAT3, HK2, ACLY, PI3K/AKT/mTOR, HIF-1*α*, SIK2, PKM2, LDHA, PGAM1	Enhanced glycolysis promotes fibroblast activation, proliferation, migration, myofibroblast differentiation, and collagen synthesis.
	Lipid metabolism	PGF2α/PGF2α receptor, PTGES, LPA/LPA1/LPA2, fatty acid metabolism, SIRT1, PPARα, CPT1A, mitochondrial ROS, Drp1, ROS/HIF-1α	Dysregulated lipid metabolism promotes fibroblast proliferation and myofibroblast differentiation, alters extracellular matrix regulation, and contributes to fibroblast activation.
	Amino acid metabolism	PI3K/AKT/mTORC1–ATF4, serine–glycine metabolism, NOX4–ARG1/OAT, ALDH18A1/P5CS, PYCR1, GLS1, TGF-*β*1/SMAD3/MAPK	Amino acid metabolic remodeling supports fibroblast activation, myofibroblast differentiation, collagen synthesis, and extracellular matrix deposition.
Alveolar epithelial cells	Glucose metabolism	HIF-1α, LDH isoforms, lactate, NAD+/NADH, ATF4–CHOP, PINK1-mediated mitochondrial autophagy, mitochondrial oxidative phosphorylation	Glycolytic and mitochondrial metabolic abnormalities are associated with AEC2 senescence, apoptosis, EMT, mitochondrial dysfunction, and impaired epithelial homeostasis.
	Lipid metabolism	ACSS3, CPT1A, FASN, PPAR-*γ*, phosphatidylcholine metabolism, cholesterol metabolism, triglyceride synthesis, SP-C, LPCAT1	Lipid metabolic dysregulation impairs surfactant homeostasis, mitochondrial function, lipid vesicle uptake, alveolar surface-tension regulation, and AEC2 regeneration, and promotes epithelial cell injury/apoptosis.
	Amino acid metabolism	Glutamine catabolism, glutamate metabolism, arginine metabolism, N*ε*-carboxymethyllysine	Altered amino acid metabolism is associated with impaired AEC2 proliferation and differentiation and abnormal epithelial repair.
Macrophages	Glucose metabolism	HK2, PKM2, LDHA, HIF-1α, Ac-SDKP, IGF2BP1–THBS1–TLR4, lactate, histone lactylation	Enhanced glycolytic reprogramming and lactate accumulation promote macrophage activation and M2/profibrotic polarization, inflammatory signaling, TGF-β1-related responses, and pulmonary fibrosis.
	Lipid metabolism	PPARG/SPP1/CD44, aldose reductase, ERK–MYC, FASN, ACC1, S1PR3, PI3K/AKT/STAT3, TREM2, sphingomyelin metabolism, CCL2	Dysregulated lipid uptake and metabolism impair fatty acid utilization and promote lipid accumulation, M2/profibrotic macrophage polarization, chemokine secretion, fibroblast activation, and extracellular matrix deposition.
	Amino acid metabolism	RIPK3, AKT–mTOR, arginine–polyamine metabolism, TGF-β1, PAD4, arginine citrullination, NETs	Altered arginine metabolism and citrullination contribute to polyamine accumulation, NET formation, M1/M2 polarization imbalance, inflammatory/profibrotic cytokine release, and fibrosis.
Endothelial cells	Glucose metabolism	LDHA, aerobic glycolysis, oxidative phosphorylation, ROCK2	Glycolytic–oxidative phosphorylation imbalance affects endothelial proliferation, migration, and alveolar-capillary barrier integrity.
	Lipid metabolism	S1PR1, sphingomyelin metabolism, phospholipid metabolism, LDLR, LDL accumulation	Altered lipid signaling and metabolism increase vascular permeability and endothelial apoptosis and contribute to profibrotic phenotypic transformation.

## 7 Pulmonary fibrosis treatment strategies that target metabolic reprogramming

### 7.1 Therapeutic strategies targeting glucose metabolism

Targeted glucose metabolism reprogramming has emerged as a key therapeutic strategy to reverse the progression of pulmonary fibrosis. As the primary effector cells in fibrosis, myofibroblasts rely heavily on aerobic glycolysis, characterized by the “Warburg effect”, during their abnormal activation; this dependency provides a clear metabolic target for drug intervention. Inhibition of key glycolytic enzyme activity is the main direction of current research. PFKFB3, as a key rate-limiting enzyme for glycolysis, plays a central role in myofibroblast activation. Studies have shown that anlotinib reduces the translation efficiency of PFKFB3 by downregulating PCBP3, thereby blocking glycolysis and reversing pulmonary fibrosis in mice (27). Similarly, inhibitors targeting the PI3K-Akt-mTOR/PFKFB3 pathway can effectively block collagen synthesis in lipopolysaccharide-induced models (30). In addition, interventions targeting the upstream link of glycolysis have also shown efficacy, such as dihydromyricetin has been shown to block glucose uptake and inhibit pulmonary fibrosis in mice by interfering with the STAT3/GLUT1 signaling pathway (29), and Tanshinone IIA down-regulates HK2 expression by inhibiting the PI3K/Akt/mTOR/HIF-1*α* axis, thereby inhibiting glycolysis and improving pulmonary fibrosis in mice (31). The intervention of hypoxic microenvironment signaling pathways is another important strategy. HIF-1*α* is a master regulator driving glycolytic switching. Dichloroacetic acid significantly alleviated bleomycin-induced pulmonary fibrosis by inhibiting PDK1 downstream of HIF-1*α* and correcting the metabolic pattern (99). This mechanism has been validated in differentiation models of pulmonary fibrosis, such as the restoration of mitochondrial function by inhibiting the ROS/HIF-1*α* axis in response to PM_2.5_ exposure-induced pulmonary fibrosis (59); PTGES has also been found to promote fibrosis by activating the HIF-1*α* pathway, and its inhibition can block this process (38). In addition, SIK2 has been shown to be an upstream driver of glycolysis in fibroblasts, and inhibitors targeting SIK2, such as YKL06-061 and faustinib, have shown significant anti-fibrotic potential (32). In addition to directly inhibiting glycolysis, intervening in metabolite transport and targeting specific cell populations also provides new perspectives. Inhibiting the MCT1/4 lactate transporter and blocking lactate efflux can promote oxidative phosphorylation and alleviate fibrosis (100). Notably, the glucose metabolism treatment strategy is cell-specific, with KD025 enhancing vascular barrier integrity by promoting oxidative phosphorylation in endothelial cells (95),while Ac-SDKP treats silicosis by reversing macrophage glycolysis reprogramming (79). In summary, the therapeutic strategy of targeting glucose metabolism is shifting from single “inhibition of glycolysis” to “metabolic rebalancing.” Existing studies have shown that the core mechanism of both small molecule inhibitors and traditional Chinese medicine monomers is to correct pathological metabolic shifts. Future treatment strategies should be targeted at the metabolic characteristics of different cell types to achieve the overall remodeling of the pulmonary fibrosis microenvironment.

### 7.2 Therapeutic strategies targeting lipid metabolism

Lipid metabolism disorders are a key feature of pulmonary fibrosis, involving fatty acid metabolism, phospholipid metabolism, and sphingolipid metabolism. In recent years, targeting key nodes of lipid metabolism has become a new direction in the development of anti-fibrotic drugs, and a variety of intervention strategies have shown therapeutic potential in preclinical models. Regulating the metabolic balance of fatty acids is a crucial direction of intervention; Pulmonary fibrosis is often characterized by decreased fatty acid oxidation and increased glycolysis. While this metabolic switch promotes ECM deposition, restoring fatty acid oxidation enhances intracellular matrix degradation pathways and inhibits fibrosis progression (42). SIRT6 mitigates bleomycin-induced pulmonary fibrosis by activating PPAR*α*-mediated lipid catabolism (101). SIRT1 improves smoking-induced lipid metabolism disorders in fibroblasts by reducing mitochondrial ROS and up-regulating PPAR*α* and CPT1a expression (46). Conversely, excessive fatty acid synthesis contributes to fibrosis. Aldose reductase promotes the expression of FASN and ACC1 by activating the ERK-MYC pathway, thereby enhancing fatty acid synthesis and driving macrophage M2 polarization. Its inhibitor, epapristat, can significantly reduce pulmonary fibrosis and has a good clinical translation prospect (88). The Drp1 inhibitor Mdivi-1 attenuates fibroblast activation by blocking mitochondrial division, suppressing the ROS/HIF-1*α* pathway, and correcting lipid metabolism reprogramming (45). TREM2 is specifically upregulated in fibrotic lung macrophages, and blocking TREM2 can reduce sphingomyelin synthesis and chemokine secretion, thereby improving pulmonary fibrosis (90). S1pr3 is overexpressed in M2 macrophages, and its inhibitor CAY10444 mitigates fibrosis by inhibiting the PI3K/Akt-Stat3 pathway by reducing M2 polarization and alleviating fibrosis (89). Maintaining lipid homeostasis in epithelial cells is also critical. Selective loss of LPCAT1 in alveolar type Ⅱ cells leads to phosphatidylcholine metabolism disorder and impaired stem cell regeneration. Drugs such as Artesunate can up-regulate LPCAT1 expression, promote AT2 cell regeneration, and reduce fibrosis (102). Activation of the SENP1-Sirt3 axis maintains AT2 cell stem cells and alleviates pulmonary fibrosis by scavenging mitochondrial reactive oxygen species and enhancing lipid metabolism (103). In summary, anti-fibrosis strategies targeting lipid metabolism include multiple aspects such as promoting fatty acid oxidation, regulating macrophage polarization, and maintaining epithelial cell homeostasis. The successful application of existing drugs such as Epalrestat and Artesunate in anti-fibrosis models demonstrates the broad prospect of “drug repurposing”.

### 7.3 Therapeutic strategies targeting amino acid metabolism

Targeting glutamine metabolism is a key measure to cut off the “fuel supply” of myofibroblasts. Mulberry pigment inhibits the differentiayion of fibroblasts into myofibroblasts by activating PPAR-*γ*, which down-regulates GLS1 expression and blocks glutamine degradation (104). On the one hand, activation of Nrf2 shifts glutamine metabolism toward glutathione synthesis, thereby reducing glutamic acid used for energy metabolism and alleviating oxidative stress (105); on the other hand, directly inhibiting GLS1 activity reduces the metabolic flux entering mitochondria, thereby blocking the supply of raw materials for collagen synthesis (20). Secondly, blocking the production of amino acids, a precursor to collagen synthesis, is another important therapeutic pathway. Studies indicate that glutamine metabolism primarily generates glycine and proline via the ALDH18A1/P5CS pathway; this biosynthetic function, rather than simple energy provision, which is essential for collagen synthesis (55). Therefore, targeting these downstream synthases offers the potential for precise intervention, and Tanshinone IIA has been shown to inhibit P5CS and PYCR1 expression, thereby blocking proline synthesis (20). In addition, the complex formed by Kindlin-2 and PYCR1 has been found to be a key regulatory point for proline synthesis, and the existing antifibrotic drugs pirfenidone and nintedanib can play a role by reducing the level of this complex, which provides a new metabolic direction for the existing drug mechanism of action (52). Trichodelphinine A has also been found to inhibit collagen deposition by targeting NOX4 to regulate the arginine-proline metabolism axis (50). Finally, targeting the amino acid metabolism microenvironment of immune cells also has therapeutic prospects. In macrophages, RIPK3 promotes the conversion of arginine to polyamines through the AKT-mTOR pathway, which drives fibrosis. Inhibiting this pathway can reverse the process (91). Circ0066187 upregulates STAT3 through sponge adsorption of miR-29b-2-5p, regulates the metabolism of various amino acids such as glutamine and proline to promote fibrosis, providing potential RNA therapeutic targets (106). In conclusion, the targeted amino acid metabolism therapy strategy has developed into a multi-target and multi-pathway approach, and natural compounds (such as mulberry pigment and Tanshinone IIA) have demonstrated promising transformation potential. In the future, we should focus on clinical translation, strengthen the validation of candidate target specificity, and explore the advantages of natural compounds to promote amino acid metabolism targeting strategies to become a new treatment direction for pulmonary fibrosis.

## 8 Conclusion

This paper systematically reviews the metabolic reprogramming characteristics of core effector cells in pulmonary fibrosis, clarifying the underlying mechanisms of abnormal reprogramming of glucose metabolism, lipid metabolism and amino acid metabolism, and their key role in driving the progression of fibrosis by regulating cell activation, apoptosis, inflammatory responses and other pathological processes. Although current studies have revealed the basic characteristics and some regulatory mechanisms of metabolic reprogramming, there are still significant limitations: most treatment strategies remain in the preclinical stage and lack human clinical trial validation; there is heterogeneity in the metabolic abnormalities of pulmonary fibrosis induced by different etiologies, and a universal treatment plan has not yet been established. The cross-regulatory network between metabolic pathways and the mechanism of metabolic interaction between cells still need to be explored in depth. Future research should focus on clinical translation, strengthen research on candidate target specificity and drug safety, explore the therapeutic advantages of natural compounds, optimize treatment strategies based on individualized metabolic characteristics, promote metabolically targeted therapies from basic research to clinical application, and provide more effective treatment options for patients with pulmonary fibrosis. The overall systemic picture of how multiple cell types interact to promote IPF pathogenesis remains missing (17). Therefore, metabolic reprogramming is regarded as an urgently needed therapeutic intervention approach, and the main causal relationship between metabolic reprogramming and IPF requires further research to clarify.

## References

[1] Jiang M Bu W Wang X Ruan J Shi W Yu S. Pulmonary fibrosis: from mechanisms to therapies. J Transl Med. (2025) 23:515. 10.1186/s12967-025-06514-2 40340941 PMC12063347

[2] Wijsenbeek M Suzuki A Maher TM. Interstitial lung diseases. Lancet. (2022) 400:769–86. 10.1016/S0140-6736(22)01052-2 35964592

[3] Gonnelli F Bonifazi M Hubbard R. Mortality trends in idiopathic pulmonary fibrosis in Europe between 2013 and 2018. Eur Respir J. (2024) 64:2302080. 10.1183/13993003.02080-2023 38871377 PMC11339406

[4] Maher TM Bendstrup E Dron L Langley J Smith G Khalid JM. Global incidence and prevalence of idiopathic pulmonary fibrosis. Respir Res. (2021) 22:197. 10.1186/s12931-021-01791-z 34233665 PMC8261998

[5] Raghu G Remy-Jardin M Myers JL Richeldi L Ryerson CJ Lederer DJ. Diagnosis of idiopathic pulmonary fibrosis. An official ATS/ERS/JRS/ALAT clinical practice guideline. Am J Respir Crit Care Med. (2018) 198:e44–68. 10.1164/rccm.201807-1255ST 30168753

[6] Mei Q Liu Z Zuo H Yang Z Qu J. Idiopathic pulmonary fibrosis: an update on pathogenesis. Front Pharmacol. (2022) 12:797292. 10.3389/fphar.2021.797292 35126134 PMC8807692

[7] Li J Zhai X Sun X Cao S Yuan Q Wang J. Metabolic reprogramming of pulmonary fibrosis. Front Pharmacol. (2022) 13:1031890. 10.3389/fphar.2022.1031890 PMC9702072 36452229

[8] Yang S Hu C Chen X Tang Y Li J Yang H. Crosstalk between metabolism and cell death in tumorigenesis. Mol Cancer. (2024) 23:71. 10.1186/s12943-024-01977-1 38575922 PMC10993426

[9] Liao M Yao D Wu L Luo C Wang Z Zhang J. Targeting the warburg effect: a revisited perspective from molecular mechanisms to traditional and innovative therapeutic strategies in cancer. Acta Pharm Sin B. (2024) 14:953–1008. 10.1016/j.apsb.2023.12.003 38487001 PMC10935242

[10] Li Z Munim MB Sharygin DA Bevis BJ Vander Heiden MG. Understanding the Warburg effect in cancer. Cold Spring Harb Perspect Med. (2025) 15:a041532. 10.1101/cshperspect.a041532 39284669 PMC12667404

[11] Zhu X Jiang L Long M Wei X Hou Y Du Y. Metabolic reprogramming and renal fibrosis. Front Med (Lausanne). (2021) 8:746920. 10.3389/fmed.2021.746920 34859009 PMC8630632

[12] Horn P Tacke F. Metabolic reprogramming in liver fibrosis. Cell Metab. (2024) 36:1439–55. 10.1016/j.cmet.2024.05.003 38823393

[13] Delgado ME Cárdenas BI Farran N Fernandez M. Metabolic reprogramming of liver fibrosis. Cells. (2021) 10:3604. 10.3390/cells10123604 34944111 PMC8700241

[14] Seo R De Guzman ACV Park S Lee JY Kang S-J. Cancer-intrinsic Cxcl5 orchestrates a global metabolic reprogramming for resistance to oxidative cell death in 3D. Cell Death Differ. (2025) 32:1200–13. 10.1038/s41418-025-01466-y 40050422 PMC12284152

[15] Drzewicka K Zasłona Z. Metabolism-driven glycosylation represents therapeutic opportunities in interstitial lung diseases. Front Immunol. (2024) 15:1328781. 10.3389/fimmu.2024.1328781 38550597 PMC10973144

[16] Yang J Pan X Xu M Li Y Liang C Liu L. Downregulation of HMGCS2 mediated AECIIs lipid metabolic alteration promotes pulmonary fibrosis by activating fibroblasts. Respir Res. (2024) 25:176. 10.1186/s12931-024-02816-z 38658970 PMC11040761

[17] Moss BJ Ryter SW Rosas IO. Pathogenic mechanisms underlying idiopathic pulmonary fibrosis. Annu Rev Pathol. (2022) 17:515–46. 10.1146/annurev-pathol-042320-030240 34813355

[18] Wang Z Chen L Huang Y Luo M Wang H Jiang Z. Pharmaceutical targeting of succinate dehydrogenase in fibroblasts controls bleomycin-induced lung fibrosis. Redox Biol. (2021) 46:102082. 10.1016/j.redox.2021.102082 34343908 PMC8342973

[19] Para R Romero F George G Summer R. Metabolic reprogramming as a driver of fibroblast activation in PulmonaryFibrosis. Am J Med Sci. (2019) 357:394–8. 10.1016/j.amjms.2019.02.003 30879738 PMC6478549

[20] Shan B Guo C Zhou H Chen J. Tanshinone IIA alleviates pulmonary fibrosis by modulating glutamine metabolic reprogramming based on [U-13C5]-glutamine metabolic flux analysis. J Adv Res. (2025) 70:531–44. 10.1016/j.jare.2024.04.029 38697470 PMC11976427

[21] Zheng C Zhang L Sun Y Ma Y Zhang Y. Alveolar epithelial cell dysfunction and epithelial-mesenchymal transition in pulmonary fibrosis pathogenesis. Front Mol Biosci. (2025) 12:1564176. 10.3389/fmolb.2025.1564176 40343260 PMC12058482

[22] Li S Li C Sun W Cao Y Qi X Zhang J. Spatially resolved metabolomics reveals metabolic heterogeneity among pulmonary fibrosis. J Mass Spectrom. (2025) 60:e5138. 10.1002/jms.5138 40264277

[23] Bueno M Calyeca J Rojas M Mora AL. Mitochondria dysfunction and metabolic reprogramming as drivers of idiopathic pulmonary fibrosis. Redox Biol. (2020) 33:101509. 10.1016/j.redox.2020.101509 32234292 PMC7251240

[24] Dai T Liang Y Li X Zhao J Li G Li Q. Targeting alveolar epithelial cell metabolism in pulmonary fibrosis: pioneering an emerging therapeutic strategy. Front Cell Dev Biol. (2025) 13:1608750. 10.3389/fcell.2025.1608750 40636669 PMC12238887

[25] Hawkins A Guttentag SH Deterding R Funkhouser WK Goralski JL Chatterjee S. A non-BRICHOS SFTPC mutant (SP-CI73T) linked to interstitial lung disease promotes a late block in macroautophagy disrupting cellular proteostasis and mitophagy. Am J Physiol Lung Cell Mol Physiol. (2015) 308:L33–47. 10.1152/ajplung.00217.2014 25344067 PMC4281696

[26] Xie N Tan Z Banerjee S Cui H Ge J Liu R-M. Glycolytic reprogramming in myofibroblast differentiation and lung fibrosis. Am J Respir Crit Care Med. (2015) 192:1462–74. 10.1164/rccm.201504-0780OC 26284610 PMC4731722

[27] Chen W Zhang J Zhong W Liu Y Lu Y Zeng Z. Anlotinib inhibits PFKFB3-driven glycolysis in myofibroblasts to reverse pulmonary fibrosis. Front Pharmacol. (2021) 12:744826. 10.3389/fphar.2021.744826 34603058 PMC8481786

[28] Cho SJ Moon J-S Lee C-M Choi AMK Stout-Delgado HW. Glucose transporter 1-dependent glycolysis is increased during aging-related lung fibrosis, and phloretin inhibits lung fibrosis. Am J Respir Cell Mol Biol. (2017) 56:521–31. 10.1165/rcmb.2016-0225OC 27997810 PMC5449513

[29] Li Z Geng J Xie B He J Wang J Peng L. Dihydromyricetin alleviates pulmonary fibrosis by regulating abnormal fibroblasts through the STAT3/p-STAT3/GLUT1 signaling pathway. Front Pharmacol. (2022) 13:834604. 10.3389/fphar.2022.834604 35359847 PMC8964100

[30] Hu X Xu Q Wan H Hu Y Xing S Yang H. PI3K-Akt-mTOR/PFKFB3 pathway mediated lung fibroblast aerobic glycolysis and collagen synthesis in lipopolysaccharide-induced pulmonary fibrosis. Lab Invest. (2020) 100:801–11. 10.1038/s41374-020-0404-9 32051533

[31] Shan B Zhou H Guo C Liu X Wu M Zhai R. Tanshinone IIA ameliorates energy metabolism dysfunction of pulmonary fibrosis using 13C metabolic flux analysis. J Pharm Anal. (2024) 14:244–58. 10.1016/j.jpha.2023.09.008 38464785 PMC10921327

[32] He J Dong R Yue H Zhang F Dou X Li X. SIK2 drives pulmonary fibrosis by enhancing fibroblast glycolysis and activation. Biomedicines. (2025) 13:1919. 10.3390/biomedicines13081919 40868174 PMC12383515

[33] Xu Q Cheng D Li G Liu Y Li P Sun W. CircHIPK3 regulates pulmonary fibrosis by facilitating glycolysis in miR-30a-3p/FOXK2-dependent manner. Int J Biol Sci. (2021) 17:2294–307. 10.7150/ijbs.57915 34239356 PMC8241722

[34] Mei S Xu Q Hu Y Tang R Feng J Zhou Y. Integrin *β*3-PKM2 pathway-mediated aerobic glycolysis contributes to mechanical ventilation-induced pulmonary fibrosis. Theranostics. (2022) 12:6057–68. 10.7150/thno.72328 36168620 PMC9475464

[35] Yang H Han M Zhang L Yao C Xu Q. CPSF6 loss mediates LDHA 3’UTR shortening to promote fibroblast glycolysis and pulmonary fibrosis. Cell Signal. (2025) 136:112165. 10.1016/j.cellsig.2025.112165 41076169

[36] Yang H Zhang L Chen G Wu Q Li M Zhang C. Vitamin D alleviates silica-induced pulmonary fibrosis partly by inhibiting STAT3/hexokinase 2-mediated fibroblast glycolysis. Toxicol Appl Pharmacol. (2025) 503:117456. 10.1016/j.taap.2025.117456 40633737

[37] Oga T Matsuoka T Yao C Nonomura K Kitaoka S Sakata D. Prostaglandin F(2alpha) receptor signaling facilitates bleomycin-induced pulmonary fibrosis independently of transforming growth factor-beta. Nat Med. (2009) 15:1426–30. 10.1038/nm.2066 19966781

[38] Lin M-H Lee Y-C Liao J-B Chou C-Y Yang Y-F. PTGES is involved in myofibroblast differentiation via HIF-1*α*-dependent glycolysis pathway. J Cell Mol Med. (2024) 28:e70157. 10.1111/jcmm.70157 39417702 PMC11484478

[39] Yung YC Stoddard NC Chun J. LPA receptor signaling: pharmacology, physiology, and pathophysiology. J Lipid Res. (2014) 55:1192–214. 10.1194/jlr.R046458 24643338 PMC4076099

[40] Tager AM LaCamera P Shea BS Campanella GS Selman M Zhao Z. The lysophosphatidic acid receptor LPA1 links pulmonary fibrosis to lung injury by mediating fibroblast recruitment and vascular leak. Nat Med. (2008) 14:45–54. 10.1038/nm1685 18066075

[41] Huang LS Fu P Patel P Harijith A Sun T Zhao Y. Lysophosphatidic acid receptor-2 deficiency confers protection against bleomycin-induced lung injury and fibrosis in mice. Am J Respir Cell Mol Biol. (2013) 49:912–22. 10.1165/rcmb.2013-0070OC 23808384 PMC3931116

[42] Sun W Zhou S Peng L Wang W Liu Y Wang T. Fatty acid oxidation-glycolysis metabolic transition affects ECM homeostasis in silica-induced pulmonary fibrosis. Adv Sci (Weinh). (2025) 12:e2407134. 10.1002/advs.202407134 39721015 PMC11831484

[43] Hwang S Chung KW. Targeting fatty acid metabolism for fibrotic disorders. Arch Pharm Res. (2021) 44:839–56. 10.1007/s12272-021-01352-4 34664210

[44] Rangarajan S Locy ML Chanda D Kurundkar A Kurundkar D Larson-Casey JL. Mitochondrial uncoupling protein-2 reprograms metabolism to induce oxidative stress and myofibroblast senescence in age-associated lung fibrosis. Aging Cell. (2022) 21:e13674. 10.1111/acel.13674 35934931 PMC9470902

[45] Tong Z Du X Zhou Y Jing F Ma J Feng Y. Drp1-mediated mitochondrial fission promotes pulmonary fibrosis progression through the regulation of lipid metabolic reprogramming by ROS/HIF-1*α*. Cell Signal. (2024) 117:111075. 10.1016/j.cellsig.2024.111075 38311302

[46] Zhang Y Li T Pan M Wang W Huang W Yuan Y. SIRT1 prevents cigarette smoking-induced lung fibroblasts activation by regulating mitochondrial oxidative stress and lipid metabolism. J Transl Med. (2022) 20:222. 10.1186/s12967-022-03408-5 35568871 PMC9107262

[47] Hamanaka RB Mutlu GM. The role of metabolic reprogramming and *de novo* amino acid synthesis in collagen protein production by myofibroblasts: implications for organ fibrosis and cancer. Amino Acids. (2021) 53:1851–62. 10.1007/s00726-021-02996-8 33963932 PMC8914507

[48] O’Leary EM Tian Y Nigdelioglu R Witt LJ Cetin-Atalay R Meliton AY. TGF-*β* promotes metabolic reprogramming in lung fibroblasts via mTORC1-dependent ATF4 activation. Am J Respir Cell Mol Biol. (2020) 63:601–12. 10.1165/rcmb.2020-0143OC PMC7605163 32668192

[49] Shin KWD Atalay MV Cetin-Atalay R O’Leary EM Glass ME Szafran JCH. mTOR signaling regulates multiple metabolic pathways in human lung fibroblasts after TGF-*β* and in pulmonary fibrosis. Am J Physiol Lung Cell Mol Physiol. (2025) 328:L215–28. 10.1152/ajplung.00189.2024 39745695 PMC12204138

[50] Liu F Yao Y Guo C Dai P Huang J Peng P. Trichodelphinine A alleviates pulmonary fibrosis by inhibiting collagen synthesis via NOX4-ARG1/TGF-*β* signaling pathway. Phytomedicine. (2024) 132:155755. 10.1016/j.phymed.2024.155755 38870750

[51] Guo L Cui C Zhang K Wang J Wang Y Lu Y. Kindlin-2 links mechano-environment to proline synthesis and tumor growth. Nat Commun. (2019) 10:845. 10.1038/s41467-019-08772-3 30783087 PMC6381112

[52] Zhang P Wang J Luo W Yuan J Cui C Guo L. Kindlin-2 acts as a key mediator of lung fibroblast activation and pulmonary fibrosis progression. Am J Respir Cell Mol Biol. (2021) 65:54–69. 10.1165/rcmb.2020-0320OC 33761308

[53] Willette RN Mangrolia P Pondell SM Lee CYW Yoo S Rudoltz MS. Modulation of oxidative phosphorylation with IM156 attenuates mitochondrial metabolic reprogramming and inhibits pulmonary fibrosis. J Pharmacol Exp Ther. (2021) 379:290–300. 10.1124/jpet.121.000811 34593558

[54] Shin KWD Atalay MV Cetin-Atalay R O’Leary EM Glass ME Houpy Szafran JC. ATF4 and mTOR regulate metabolic reprogramming in TGF-*β*-treated lung fibroblasts. bioRxiv (2024) 10.1101/2024.06.12.598694

[55] Hamanaka RB O’Leary EM Witt LJ Tian Y Gökalp GA Meliton AY. Glutamine metabolism is required for collagen protein synthesis in lung fibroblasts. Am J Respir Cell Mol Biol. (2019) 61:597–606. 10.1165/rcmb.2019-0008OC 30973753 PMC6827066

[56] Bernard K Logsdon NJ Benavides GA Sanders Y Zhang J Darley-Usmar VM. Glutaminolysis is required for transforming growth factor-*β*1-induced myofibroblast differentiation and activation. J Biol Chem. (2018) 293:1218–28. 10.1074/jbc.RA117.000444 29222329 PMC5787800

[57] Yan P Liu J Li Z Wang J Zhu Z Wang L. Glycolysis reprogramming in idiopathic pulmonary fibrosis: unveiling the mystery of lactate in the lung. Int J Mol Sci. (2024) 25:315. 10.3390/ijms25010315 PMC10779333 38203486

[58] Zhu W Tan C Zhang J. Alveolar epithelial type 2 cell dysfunction in idiopathic pulmonary fibrosis. Lung. (2022) 200:539–47. 10.1007/s00408-022-00571-w 36136136

[59] Niu H Guo F Li W. Stibene glucoside prevents PM2.5 caused pulmonary fibrosis by pseudo hypoxia, autophagy and NF-*κ*B signal pathways. Int Immunopharmacol. (2025) 156:114674. 10.1016/j.intimp.2025.114674 40262249

[60] Newton DA Lottes RG Ryan RM Spyropoulos DD Baatz JE. Dysfunctional lactate metabolism in human alveolar type II cells from idiopathic pulmonary fibrosis lung explant tissue. Respir Res. (2021) 22:278. 10.1186/s12931-021-01866-x 34711218 PMC8554831

[61] Kottmann RM Kulkarni AA Smolnycki KA Lyda E Dahanayake T Salibi R. Lactic acid is elevated in idiopathic pulmonary fibrosis and induces myofibroblast differentiation via pH-dependent activation of transforming growth factor-*β*. Am J Respir Crit Care Med. (2012) 186:740–51. 10.1164/rccm.201201-0084OC 22923663 PMC3480515

[62] Massip-Copiz MM Valdivieso ÁG Clauzure M Mori C Asensio CJA Aguilar MÁ. Epidermal growth factor receptor activity upregulates lactate dehydrogenase A expression, lactate dehydrogenase activity, and lactate secretion in cultured IB3-1 cystic fibrosis lung epithelial cells. Biochem Cell Biol. (2021) 99:476–87. 10.1139/bcb-2020-0522 33481676

[63] Sun Z He W Meng H Ji Z Qu J Yu G. Lactate activates ER stress to promote alveolar epithelial cells apoptosis in pulmonary fibrosis. Respir Res. (2024) 25:401. 10.1186/s12931-024-03016-5 39522031 PMC11550544

[64] Larson-Casey JL He C Carter AB. Mitochondrial quality control in pulmonary fibrosis. Redox Biol. (2020) 33:101426. 10.1016/j.redox.2020.101426 31928788 PMC7251238

[65] Bueno M Lai Y-C Romero Y Brands J St. Croix CM Kamga C. PINK1 deficiency impairs mitochondrial homeostasis and promotes lung fibrosis. J Clin Invest. (2015) 125:521–38. 10.1172/JCI74942 25562319 PMC4319413

[66] Xiao H Peng L Jiang D Liu Y Zhu L Li Z. IL-17A promotes lung fibrosis through impairing mitochondrial homeostasis in type II alveolar epithelial cells. J Cell Mol Med. (2022) 26:5728–41. 10.1111/jcmm.17600 36308405 PMC9667517

[67] Roque W Romero F. Cellular metabolomics of pulmonary fibrosis, from amino acids to lipids. Am J Physiol Cell Physiol. (2021) 320:C689–95. 10.1152/ajpcell.00586.2020 33471621 PMC8163573

[68] Agudelo CW Samaha G Garcia-Arcos I. Alveolar lipids in pulmonary disease. A review. Lipids Health Dis. (2020) 19:122. 10.1186/s12944-020-01278-8 32493486 PMC7268969

[69] Chung K-P Hsu C-L Fan L-C Huang Z Bhatia D Chen Y-J. Mitofusins regulate lipid metabolism to mediate the development of lung fibrosis. Nat Commun. (2019) 10:3390. 10.1038/s41467-019-11327-1 31358769 PMC6662701

[70] Wang L Yuan H Li W Yan P Zhao M Li Z. ACSS3 regulates the metabolic homeostasis of epithelial cells and alleviates pulmonary fibrosis. Biochim Biophys Acta Mol Basis Dis. (2024) 1870:166960. 10.1016/j.bbadis.2023.166960 37979225

[71] Shin H Park S Hong J Baek A-R Lee J Kim D-J. Overexpression of fatty acid synthase attenuates bleomycin induced lung fibrosis by restoring mitochondrial dysfunction in mice. Sci Rep. (2023) 13:9044. 10.1038/s41598-023-36009-3 37270622 PMC10239437

[72] Kokuho N Terasaki Y Kunugi S Saito Y Urushiyama H Terasaki M. Analyses of alveolar epithelial injury via lipid-related stress in mammalian target of rapamycin inhibitor-induced lung disease. Lab Invest. (2019) 99:853–65. 10.1038/s41374-018-0158-9 30728465

[73] Mulugeta S Nureki S-I Beers MF. Lost after translation: insights from pulmonary surfactant for understanding the role of alveolar epithelial dysfunction and cellular quality control in fibrotic lung disease. Am J Physiol Lung Cell Mol Physiol. (2015) 309:L507–525. 10.1152/ajplung.00139.2015 26186947 PMC4572416

[74] Shi X Chen Y Shi M Gao F Huang L Wang W. The novel molecular mechanism of pulmonary fibrosis: insight into lipid metabolism from reanalysis of single-cell RNA-seq databases. Lipids Health Dis. (2024) 23:98. 10.1186/s12944-024-02062-8 38570797 PMC10988923

[75] Wang S Li X Ma Q Wang Q Wu J Yu H. Glutamine metabolism is required for alveolar regeneration during lung injury. Biomolecules. (2022) 12:728. 10.3390/biom12050728 35625656 PMC9138637

[76] Shaghaghi H Para R Tran C Roman J Ojeda-Lassalle Y Sun J. Glutamine restores mitochondrial respiration in bleomycin-injured epithelial cells. Free Radic Biol Med. (2021) 176:335–44. 10.1016/j.freeradbiomed.2021.10.006 34634441 PMC9121335

[77] Machahua C Montes-Worboys A Llatjos R Escobar I Dorca J Molina-Molina M. Increased AGE-RAGE ratio in idiopathic pulmonary fibrosis. Respir Res. (2016) 17:144. 10.1186/s12931-016-0460-2 27816054 PMC5097848

[78] Sun W Ren J Jia Z Liang P Li S Song M. Untargeted metabolomics reveals alterations of rhythmic pulmonary metabolism in IPF. Metabolites. (2023) 13:1069. 10.3390/metabo13101069 37887394 PMC10608701

[79] Mao N Yang H Yin J Li Y Jin F Li T. Glycolytic reprogramming in silica-induced lung macrophages and silicosis reversed by ac-SDKP treatment. Int J Mol Sci. (2021) 22:10063. 10.3390/ijms221810063 34576239 PMC8465686

[80] Xie N Cui H Ge J Banerjee S Guo S Dubey S. Metabolic characterization and RNA profiling reveal glycolytic dependence of profibrotic phenotype of alveolar macrophages in lung fibrosis. Am J Physiol Lung Cell Mol Physiol. (2017) 313:L834–44. 10.1152/ajplung.00235.2017 28798256 PMC5792180

[81] Lu Y Mu M RenChen X Wang W Zhu Y Zhong M. 2-Deoxy-D-glucose Ameliorates inflammation and fibrosis in a silicosis mouse model by inhibiting hypoxia-inducible factor-1*α* in alveolar macrophages. Ecotoxicol Environ Saf. (2024) 269:115767. 10.1016/j.ecoenv.2023.115767 38039851

[82] Liu Z Liu W Wei H Ping Y Yu Z Dong Z. Elevated lactate production exacerbates PM2.5-induced pulmonary fibrosis by stabilizing TGF-*β*1. J Adv Res. (2026) 83:299–314. 10.1016/j.jare.2025.07.057 40759348 PMC13131523

[83] Hu Y Yang L Huang L Zeng C Ren S. M6a reader IGF2BP1 facilitates macrophage glycolytic metabolism and fibrotic phenotype by stabilizing THBS1 mRNA to promote pulmonary fibrosis. Cell Mol Life Sci. (2025) 82:157. 10.1007/s00018-025-05673-1 40220148 PMC11993514

[84] Li J Zeng G Zhang Z Wang Y Shao M Li C. Urban airborne PM2.5 induces pulmonary fibrosis through triggering glycolysis and subsequent modification of histone lactylation in macrophages. Ecotoxicol Environ Saf. (2024) 273:116162. 10.1016/j.ecoenv.2024.116162 38458067

[85] Xu Q Mei S Nie F Zhang Z Feng J Zhang J. The role of macrophage-fibroblast interaction in lipopolysaccharide-induced pulmonary fibrosis: an acceleration in lung fibroblast aerobic glycolysis. Lab Invest. (2022) 102:432–9. 10.1038/s41374-021-00701-7 34775492

[86] Zhang Y Fu J Han Y Feng D Yue S Zhou Y. Two-pore-domain potassium channel TREK-1 mediates pulmonary fibrosis through macrophage M2 polarization and by direct promotion of fibroblast differentiation. Biomedicines. (2023) 11:1279. 10.3390/biomedicines11051279 37238950 PMC10215911

[87] Li G Zhang Y Jiang H Wu X Hao Y Su Y. PPARG/SPP1/CD44 signaling pathway in alveolar macrophages: mechanisms of lipid dysregulation and therapeutic targets in idiopathic pulmonary fibrosis. Heliyon. (2025) 11:e41628. 10.1016/j.heliyon.2025.e41628 39866448 PMC11761845

[88] Li J Zhang Q Zhao P Mei X Shi Y Shao W. Targeting aldose reductase in pulmonary fibrosis: blocking *de novo* fatty acid synthesis halts pro-fibrotic M2 polarization. Int J Biol Macromol. (2026) 337:149590. 10.1016/j.ijbiomac.2025.149590 41380895

[89] Qiu H Liu J You J Zhou O Hao C Shu Y. Inhibition of sphingosine 1-phosphate receptor 3 ameliorates bleomycin-induced pulmonary fibrosis by suppressing macrophage M2 polarization. Genes Dis. (2025) 12:101244. 10.1016/j.gendis.2024.101244 40092491 PMC11907442

[90] Gu X Kang H Cao S Tong Z Song N. Blockade of TREM2 ameliorates pulmonary inflammation and fibrosis by modulating sphingolipid metabolism. Transl Res. (2025) 275:1–17. 10.1016/j.trsl.2024.10.002 39490681

[91] Yang T Li X Lei S Li Q Zhang Y Hui Z. RIPK3 orchestrates scar-associated macrophage dysfunction to drive pulmonary fibrosis. Adv Sci (Weinh). (2026) 13:e19404. 10.1002/advs.202519404 41709822 PMC13104126

[92] Panda B Chilvery S Devi P Kalmegh R Godugu C. Inhibition of peptidyl arginine deiminase-4 ameliorated pulmonary fibrosis via modulating M1/M2 polarisation of macrophages. Life Sci. (2025) 362:123354. 10.1016/j.lfs.2024.123354 39755270

[93] Callaghan NI Davenport Huyer L. Altered metabolism in idiopathic pulmonary fibrosis. J Cell Physiol. (2025) 240:e70112. 10.1002/jcp.70112 41424408 PMC12720235

[94] Parra-Bonilla G Alvarez DF Al-Mehdi A-B Alexeyev M Stevens T. Critical role for lactate dehydrogenase A in aerobic glycolysis that sustains pulmonary microvascular endothelial cell proliferation. Am J Physiol Lung Cell Mol Physiol. (2010) 299:L513–522. 10.1152/ajplung.00274.2009 20675437 PMC2957419

[95] Lee JY Stevens RP Kash M Zhou C Koloteva A Renema P. KD025 shifts pulmonary endothelial cell bioenergetics and decreases baseline lung permeability. Am J Respir Cell Mol Biol. (2020) 63:519–30. 10.1165/rcmb.2019-0435OC 32628869 PMC7528923

[96] Knipe RS Spinney JJ Abe EA Probst CK Franklin A Logue A. Endothelial-specific loss of sphingosine-1-phosphate receptor 1 increases vascular permeability and exacerbates bleomycin-induced pulmonary fibrosis. Am J Respir Cell Mol Biol. (2022) 66:38–52. 10.1165/rcmb.2020-0408OC 34343038 PMC8803357

[97] Shi X Chen Y Liu Q Mei X Liu J Tang Y. LDLR dysfunction induces LDL accumulation and promotes pulmonary fibrosis. Clin Transl Med. (2022) 12:e711. 10.1002/ctm2.711 35083881 PMC8792399

[98] Kulkarni YM Dutta S Iyer AKV Wright CA Ramesh V Kaushik V. A lipidomics approach to identifying key lipid species involved in VEGF-inhibitor mediated attenuation of bleomycin-induced pulmonary fibrosis. Proteomics Clin Appl. (2018) 12:e1700086. 10.1002/prca.201700086 29283216 PMC6368219

[99] Goodwin J Choi H Hsieh M- Neugent ML Ahn J-M Hayenga HN. Targeting hypoxia-inducible factor-1*α*/pyruvate dehydrogenase kinase 1 axis by dichloroacetate suppresses bleomycin-induced pulmonary fibrosis. Am J Respir Cell Mol Biol. (2018) 58:216–31. 10.1165/rcmb.2016-0186OC 28915065 PMC5805994

[100] Ziehr DR Li F Mark Parnell K Krah NM Leahy KJ Guillermier C. Lactate transport inhibition therapeutically reprograms fibroblast metabolism in experimental pulmonary fibrosis. bioRxiv (2024) 10.1101/2024.04.25.591150

[101] He J Yu C Shen Y Huang J Zhou Y Gu J. Sirtuin 6 ameliorates bleomycin-induced pulmonary fibrosis via activation of lipid catabolism. J Cell Physiol. (2024) 239:e31027. 10.1002/jcp.31027 37099691

[102] Rabata A Qiao Y Li W Huang H Yu S Zhang X. Phosphatidylcholine Metabolism Controls Alveolar Progenitor Renewal and Pulmonary Fibrosis bioRxiv (2025) 10.1101/2025.09.26.678894

[103] Zhang M Lin X He J Zuo Y Fan Q Agida I. SENP1-Sirt3 axis regulates type II alveolar epithelial cell activity to confer resistance against oxidative damage in lung tissue. Redox Biol. (2025) 85:103752. 10.1016/j.redox.2025.103752 40633428 PMC12274925

[104] Miao Y Geng Y Yang L Zheng Y Dai Y Wei Z. Morin inhibits the transformation of fibroblasts towards myofibroblasts through regulating “PPAR-*γ*-glutaminolysis-DEPTOR” pathway in pulmonary fibrosis. J Nutr Biochem. (2022) 101:108923. 10.1016/j.jnutbio.2021.108923 34843935

[105] An L Peng L-Y Sun N-Y Yang Y-L Zhang X-W Li B. Tanshinone IIA activates nuclear factor-erythroid 2-related factor 2 to restrain pulmonary fibrosis via regulation of redox homeostasis and glutaminolysis. Antioxid Redox Signal. (2019) 30:1831–48. 10.1089/ars.2018.7569 30105924

[106] Liu B Liu W Li H Zhai N Lv C Song X. Circ0066187 promotes pulmonary fibrogenesis through targeting STAT3-mediated metabolism signal pathway. Cell Mol Life Sci. (2025) 82:79. 10.1007/s00018-025-05613-z 39969586 PMC11839971

[107] Jiang S Li H Zhang L Mu W Zhang Y Chen T. Generic diagramming platform (GDP): a comprehensive database of high-quality biomedical graphics. Nucleic Acids Res. (2025) 53(D1):D1670–6. 10.1093/nar/gkae973 39470721 PMC11701665

